# A Modular Active–Reactive AAS Architecture for Mass-Customized Production: Validation on an Industrial-Grade Robotic Screwdriving Demonstrator

**DOI:** 10.3390/s26175523

**Published:** 2026-08-31

**Authors:** Allan Roberto Amorim da Silva, Fabio de Sousa Cardoso, Miguel Angel Orellana Postigo, Israel Gondres Torné, Yoko Lucila Takano, Luiz Pedro Araújo de Almeida, Maicon Wellington Pantoja de Souza, Afonso Henrique Torres Lucas

**Affiliations:** 1 TPV Technology, Manaus 69058-830, AM, Brazil; 2 Escola Superior de Tecnologia (EST), Universidade do Estado do Amazonas (UEA), Manaus 69050-020, AM, Brazil; fcardoso@uea.edu.br (F.d.S.C.); mpostigo@uea.edu.br (M.A.O.P.); itorne@uea.edu.br (I.G.T.); ahtl.eng22@uea.edu.br (A.H.T.L.); 3 CyberLabs-UEA, Universidade do Estado do Amazonas (UEA), Manaus 69050-020, AM, Brazil; yoko.janzen@cyberlabs-uea.org (Y.L.T.); luiz.almeida@cyberlabs-uea.org (L.P.A.d.A.); maicon.souza@cyberlabs-uea.org (M.W.P.d.S.)

**Keywords:** asset administration shell, mass customization, modular architecture, ActiveSkills, ActiveSteps, digital twin, Industry 4.0, microservices, robotic screwdriving

## Abstract

Mass-customized production introduces variants faster than certified asset models can be rebuilt. This feasibility study presents an active–reactive architecture for type-3 Asset Administration Shells (AASs) in which two submodel templates separate the reactive layer from independently deployable decision services. On the requester side, *ActiveSteps* represents a variant’s process sequence and references the capability each step needs, so a product’s AAS becomes a type-3 on demand. On the provider side, *ActiveSkills* maps offered skills to the capabilities that realize them, so one provider-core image serves every resource and only its domain services are scenario-specific. The type-2 to type-3 upgrade adds these submodels to both sides and costs one restart of the provider alone; subsequent rollouts needed none, so consumers were not interrupted. Evaluation used a university robotic screwdriving demonstrator, not a live production line. Of 104 calls for proposal, 102 were answered at a median of 4 s. Median requester activation was 8 s over 100 activations. Capability checking over 454 balanced cases with five repetitions reached an F1 of 89.6% and a 10.8% false-positive rate. An evaluation costs USD $7.0\times 10^{-4}$. These results support feasibility for asynchronous coordination in the evaluated cell; scalability and cross-vendor interoperability were untested.

## 1. Introduction

Mass-customized production requires frequent introduction of product variants without sacrificing throughput or traceability. This raises a deployment challenge: the digitalization effort invested in reactive Asset Administration Shells (AASs) must be preserved, while the active decision layer evolves rapidly to orchestrate new process plans and resources. The AAS remains a core element of the Reference Architectural Model Industrie 4.0 because it offers standardized asset representation and structured information exchange [1,2], yet most deployments still emphasize passive or reactive behavior [3,4].

Recent studies relate AASs and digital twins [5,6], explore proactive AAS concepts for order-driven production and type-3 implementations [7,8,9,10], and refine capability–skill–service models for flexible manufacturing [11,12]. Other work investigates semantic service composition and industrial-agent approaches [13,14,15] and feasibility checking in AAS-enabled work cells [16]. However, few works show how to operationalize proactive behavior for mass customization while keeping reactive AAS assets stable. This gap is sharper when the decision layer must be redeployed quickly across heterogeneous stacks and still interoperate with canonical AAS submodels.

This paper proposes a modular active–reactive architecture for type-3 AASs aimed at mass-customized production. The reactive layer holds the authoritative digital representation of each asset. The active layer is packaged as modular microservices that can be deployed, updated, or replaced with no structural change to the reactive layer. Decoupling rests on two submodels: *ActiveSkills*, which lists the skills a resource offers and references the capability submodel that implements each one, and *ActiveSteps*, which orders the process steps of a product variant and references the capability each step needs. Neither submodel carries process parameters, endpoints or policies. Both reference execution-oriented submodels in the reactive shell without modifying them, so decision logic can be iterated without disturbing certified asset descriptions.

The paper reports a validation carried out on an industrial-grade robotic screwdriving demonstrator built at the Universidade do Estado do Amazonas. The demonstrator reproduces a finished-product assembly station of the type deployed in the Manaus industrial district, with three six-axis collaborative robots, dedicated screwing jigs, screw dispensers, and a linear transport system. It is not a live production line, so the reported measurements characterise the architecture under production-like conditions and not the output of an operating factory. The experiment exercised requester–provider bidding, automated active-layer deployment, and AI-assisted capability checking, with no modification to the reactive layer. The primary contribution is the architecture itself: a deployable active–reactive organization for mass customization that preserves reactive AAS assets and lets the active decision layer evolve independently. The deployment pipeline, *ActiveSkills*/*ActiveSteps* decoupling strategy, and demonstrator measurements are supporting contributions used to validate the architectural proposal.

## 2. Materials and Methods

### 2.1. Research Design, Objectives and Hypotheses

The study followed an applied research strategy oriented to mass-customized production. Its objective was to define and validate an active–reactive organization of the type-3 AAS in which the contract between the two layers is expressed as two AAS submodels, and to determine what each submodel provides on each side of the negotiation.

The sense in which the reactive layer is preserved needs to be made precise, because the phrase covers three distinct properties and the two layers do exchange data at run time.

**Additive upgrade.** An asset that already exposes a reactive (type-2) AAS becomes type-3 by addition. Its existing submodels keep their structure and their consumers. The upgrade inserts a pair of submodels on each side: on the requester side an *ActiveSteps* submodel and the submodel declaring the capability the product needs, which *ActiveSteps* references; on the provider side an *ActiveSkills* submodel and the submodel declaring the capability the resource offers. The decision and execution services that read them are deployed alongside. No existing submodel is refactored. The cost of the upgrade is not symmetric. On the provider side, inserting these submodels and attaching the active layer calls for one restart of the reactive part, because the reactive AAS of a resource loads its submodel set at start-up, so a new submodel becomes visible only after a restart. That is a one-time cost per resource. On the requester side no restart is needed: product submodels are inserted through the standardized API of the BaSyx server, which keeps serving while the model is updated.

**Structural stability.** Following the upgrade, active-layer rollouts do not introduce structural changes to existing submodels. No submodel is removed or retyped; no submodel element is added, removed, or retyped; and no semanticId is modified. Element *values*, however, are intentionally updated. The active components of both the requester and the provider write the state generated by their decision algorithms back to their respective reactive AASs. A representative example is the update of the StepStatus property within the *ActiveSteps* submodel. This state write-back preserves process observability for any standards-compliant AAS consumer through the canonical AAS interfaces, without requiring the consumer to be aware of the existence or implementation of the active layer.

**Service continuity.** Once an AAS is type-3, the active layer can be updated, replaced or rolled back with no restart of the reactive layer, so consumers reading the reactive AAS are not interrupted. This is the operational payoff of the decoupling. Decision logic hosted inside an AAS server cannot be changed without restarting that server, and every consumer of that server pays for the change.

The two sides of the negotiation have different needs, and this work treats them separately. On the requester side, product variants come into existence in the cyber world on demand: a product AAS becomes a type-3 at the moment it enters the line and ceases to exist (as type-3) once its order is complete, so instantiation cost and deployment latency dominate. On the provider side, resources are long-lived and are not redeployed at that rate. What matters there is that the active part does not depend on which submodels implement each function, so that it can be generated instead of written.

**H1 (requester side).**  *Product variants can be maintained as modeling files in a standard AAS server and their active part instantiated on demand, so that introducing a variant needs only the deployment of an active-layer package and its ActiveSteps submodel, with no structural change to the reactive submodels already held in the server. H1 is assessed through the active-layer deploy time $t_{deploy}$, the deploy success rate $S_{deploy}$, and the record of submodels added and submodels refactored during the upgrade of the demonstrator assets.*

**H2 (provider side).**  *The ActiveSkills submodel makes the provider core agnostic to which capability and skill submodels implement each function, so that one provider-core image is reused across resources and what a scenario needs of its own is confined to the services the core calls. H2 is assessed by operating one provider-core image, instantiated once per robot, alongside the capability, feasibility and working services of the screwing scenario, and by updating those working services with no restart of the reactive layer.*

**H3 (both sides).**  *The resulting requester–provider negotiation sustains latency and decision quality compatible with asynchronous production coordination. H3 is assessed through $t_{cfp}$, $t_{accept}$ and $t_{cycle}$, and through the structural conformity and F1 of the capability-checking service.*

The validation was conducted in an industrial-level robotic screwdriving demonstrator at the Universidade do Estado do Amazonas. The scope covers asynchronous negotiation and orchestration for finished-product assembly. One consequence of H2 lies outside the evaluation scope of this paper and is stated as an implication, not a result: an active layer that is agnostic to its implementation submodels can be produced by a modeling tool, and the provider core is being incorporated as a component of a type-3 AAS creation and deployment tool in ongoing work. Cybersecurity hardening, large-scale scheduling, and enterprise integration are acknowledged as future work.

### 2.2. Active–Reactive Organization of the Type-3 AAS

The architecture organizes the type-3 AAS into two layers. The reactive layer keeps the canonical description of each asset, its execution submodels, and standardized access through HTTP, MQTT bridges, or OPC UA [2,17,18]. The active layer packages negotiation, capability checking, and orchestration logic as microservices that can be rolled out or rolled back independently, following the broader move toward containerized proactive AAS implementations [19].

Two submodels define the contract between layers. The *ActiveSkills* submodel declares the skills a resource offers and references the capability submodel that implements each one. The *ActiveSteps* submodel encodes the orchestration of process steps for specific product variants, referencing execution submodels but not modifying them. This separation enables automated deployment of new skills and step sequences with no structural change to the reactive layer, which preserves its certification trail (Section 2.1).

In the present work, the automated deployment pipeline is provided by AASistant, a digital twin orchestration platform developed at TPV and aligned with the IDTA-01001-3-0 specification, which supports both reactive (type-2) and active (type-3) AASs. AASistant exposes an HTTP gateway through which active-layer packages are registered and dispatches deploy commands over an AMQP broker to per-host deploy workers; these workers pull the corresponding container images from a private registry, fetch modeling and context files, and instantiate the provider and requester bundles as Docker containers on the target deploy host. This mechanism materializes, at the engineering level, the independence between the active and reactive layers postulated by the architecture: a new ActiveSkills or ActiveSteps package is rolled out by AASistant without any change to the underlying reactive AAS.

### 2.3. Active-Layer Deployment Pipeline (AASistant)

In AASistant the deployment of an active-layer bundle follows a short HTTP–AMQP pipeline in which the gateway never talks to Docker directly. A client request reaches the gateway-api, which publishes a deploy command to a RabbitMQ broker; on the target host, a deploy-service worker consumes the command and materializes the bundle as Docker containers, replying asynchronously with a success or error event. This indirection lets each deploy host be managed independently and tolerates the variable image-pull latency typical of industrial environments, which would otherwise exceed a synchronous HTTP timeout.

**Figure 1:**
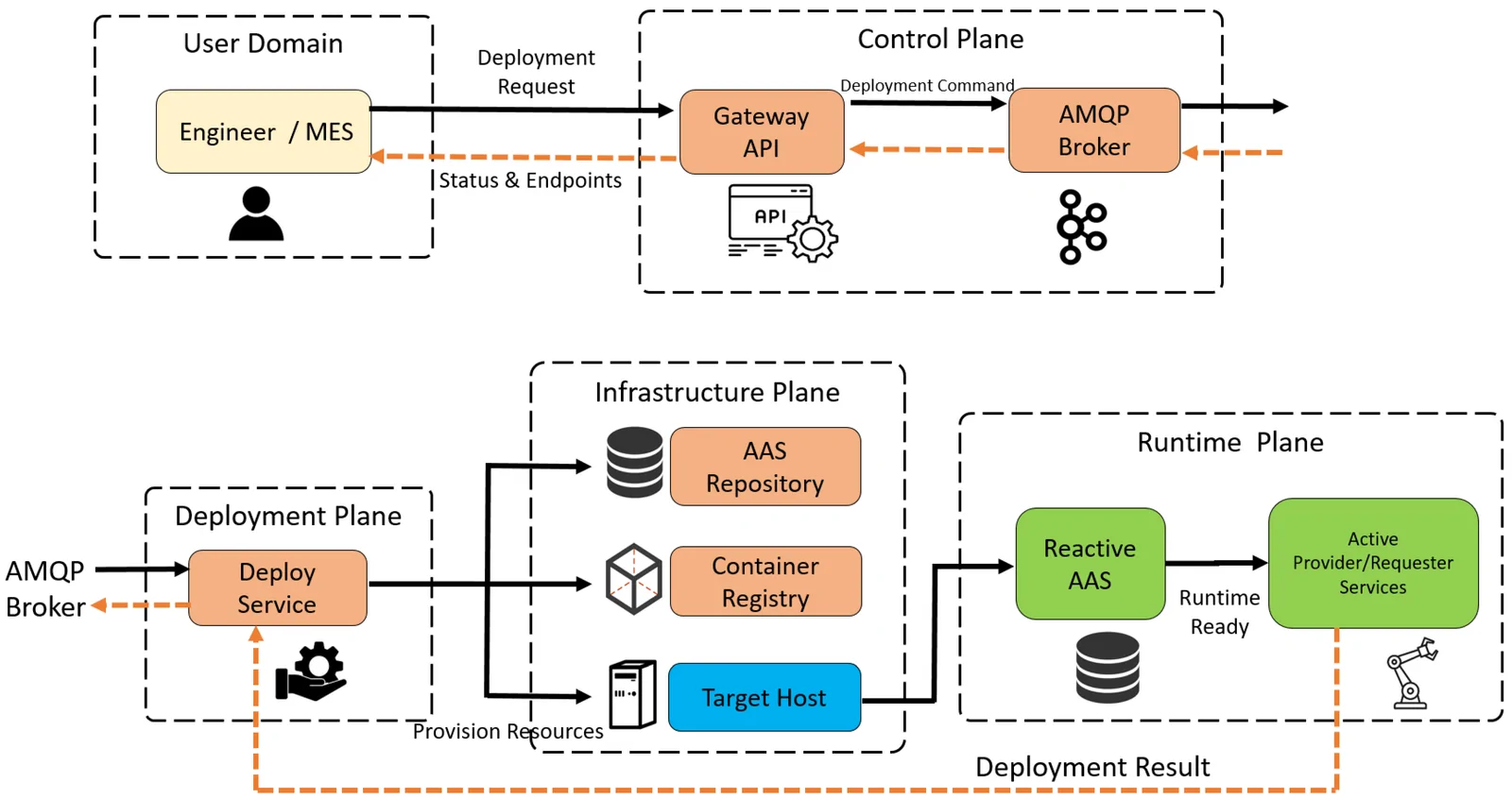
Deployment of an active-layer bundle in AASistant. The gateway-api receives the request and publishes a deploy command to RabbitMQ; a deploy-service worker on the target host materializes the reactive layer (AAS shell and MongoDB) and then the active bundle, which differs only in the set of active services started (provider or requester).

Provider and requester bundles share the same reactive prelude—the AAS shell and its MongoDB—and differ only in the active services that are then started. For the provider, the deploy-service starts the capability, feasibility and working services before provider-core, since the latter consumes them when answering Calls for Proposal. For the requester, the active phase is reduced to the evaluation-service and requester-core containers. Figure 1 summarizes both flows as a single exchange.

### 2.4. ActiveSkills and ActiveSteps Strategy

The decoupling strategy keeps proactive behavior declarative and portable, and it does so by keeping both submodels free of behavior. Neither carries process parameters, endpoints or policies. Each one is an indirection.

The *ActiveSteps* submodel holds the ordered steps of a product variant. Each step references the capability submodel that the step requires, declares whether the step may be put out to bid, carries the execution status written back by the active layer, and collects the cost attributes accumulated for that step. The *ActiveSkills* submodel holds the skills a resource offers and, for each skill, references the capability submodel that implements it. Process parameters such as torque bounds, depth limits and setup coordinates are held in the referenced capability submodels and not in either of these two.

This is what makes the active layer replaceable. A provider core resolves a reference and reads the capability submodel it finds, so it needs no knowledge of which capabilities exist and one image is reused across resources. What a scenario does need are the services that implement its capability check, its feasibility check and its execution, and those are deployed alongside the core instead of compiled into it. Endpoints, retry and timeout policies, and QoS targets belong to the deployment.

Neither submodel is IDTA-standardized. Both were defined for this architecture and are documented internally. They were built as submodel templates following the modeling rules of the IDTA metamodel, with element multiplicity declared through template qualifiers, and they are instantiated per asset under the standard instantiation rules. Concept descriptions reuse IEC CDD or ECLASS definitions where a matching property exists, and a local concept description is created only where none does. Because both submodels are added and never substituted, a client that reads the reactive AAS through the canonical API is unaffected by their presence. Interoperability of the active layer across vendors would call for these two submodels to be standardized, which the present work does not claim.

**Figure 2:**
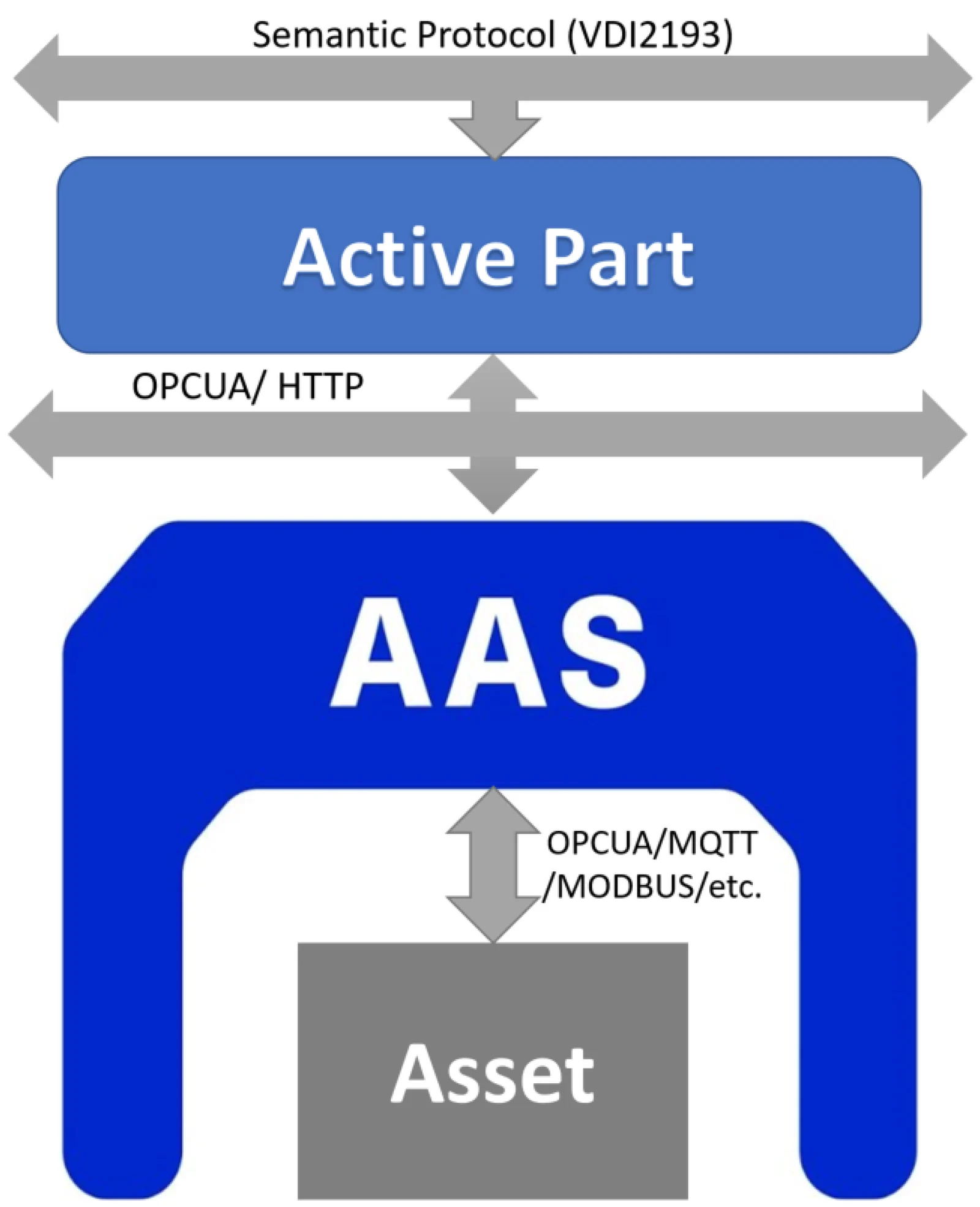
Implementation view of the type-3 AAS, where the active layer is deployed as an external microservice while the reactive layer remains a standards-aligned AAS.

Table 1 specifies both templates. Both reference execution-oriented submodels in the reactive AAS but never alter them, which preserves certification and interoperability. When a new variant is introduced, only the active-layer package and its *ActiveSteps* are deployed; the reactive shell receives no structural change (Figure 2).

### 2.5. Requester–Provider Interaction Model

The interaction model follows the Product–Process–Resource logic. The product variant acts as requester, publishing a call for proposal (CFP) that specifies screwdriving requirements (torque, sequence, fixture constraints). Providers are the robot cell and auxiliary devices exposing reactive AASs. Provider discovery happens indirectly through the proposals each provider emits in response to the CFP. Selection considers technical fit and estimated cycle time. This setup reflects shared-production literature [8,11,12] while targeting mass customization.

The requester side was split into a core service and a backend. The requester core handled CFP emission, communication state, and asynchronous message exchange. The requester backend handled evaluation logic and provider selection. On the provider side, the architecture was split into a provider core, a capability service, a feasibility service, and a domain-specific execution service. MQTT was used to carry the semantic interaction in the Industrie 4.0 language (VDI 2193), while HTTP and OPC UA were used to query and expose reactive asset information.

### 2.6. Robotic Screwdriving Demonstrator

**Figure 3:**
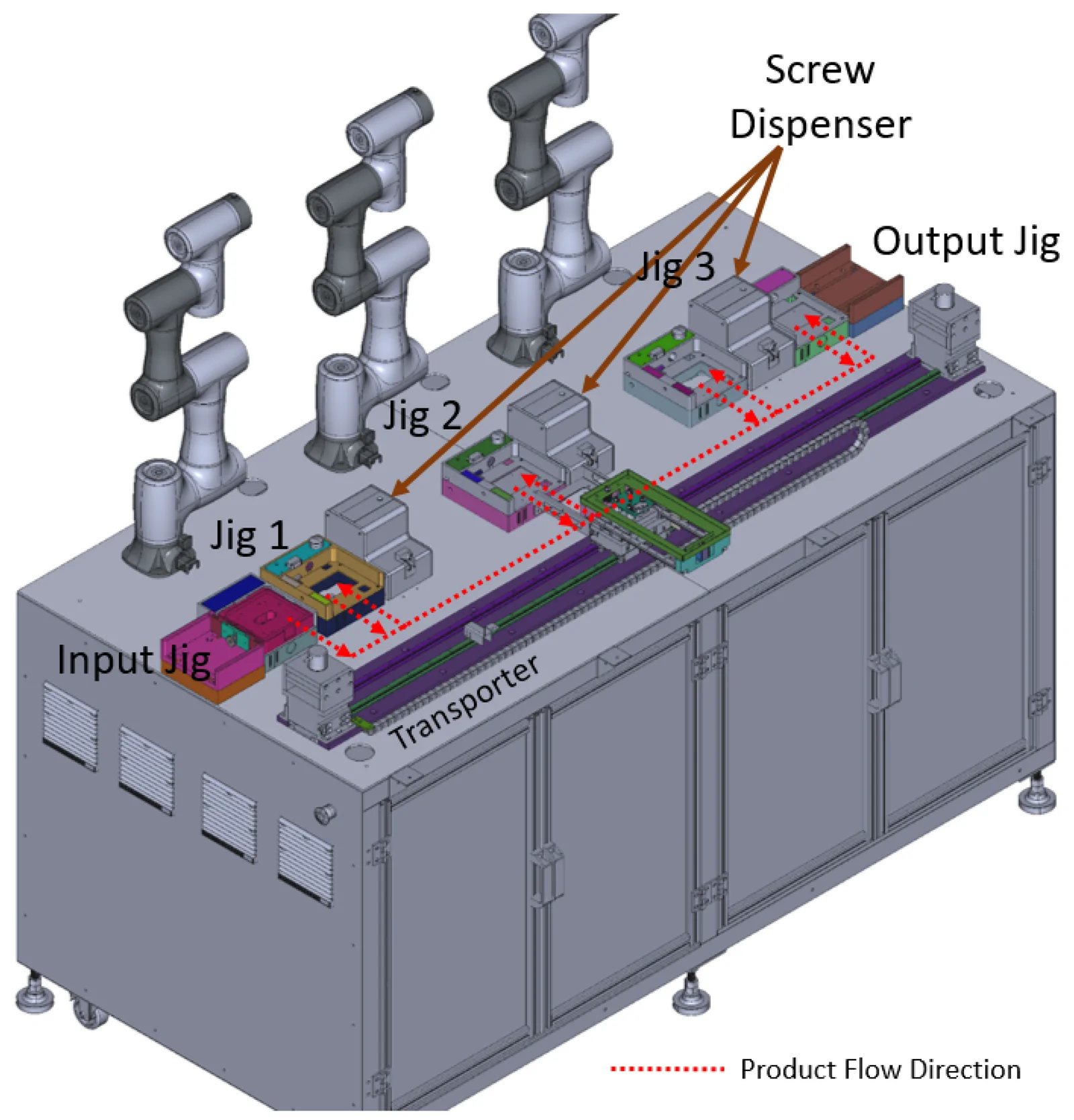
Overall view of the robotic screwdriving demonstrator used for validation.

**Figure 4:**
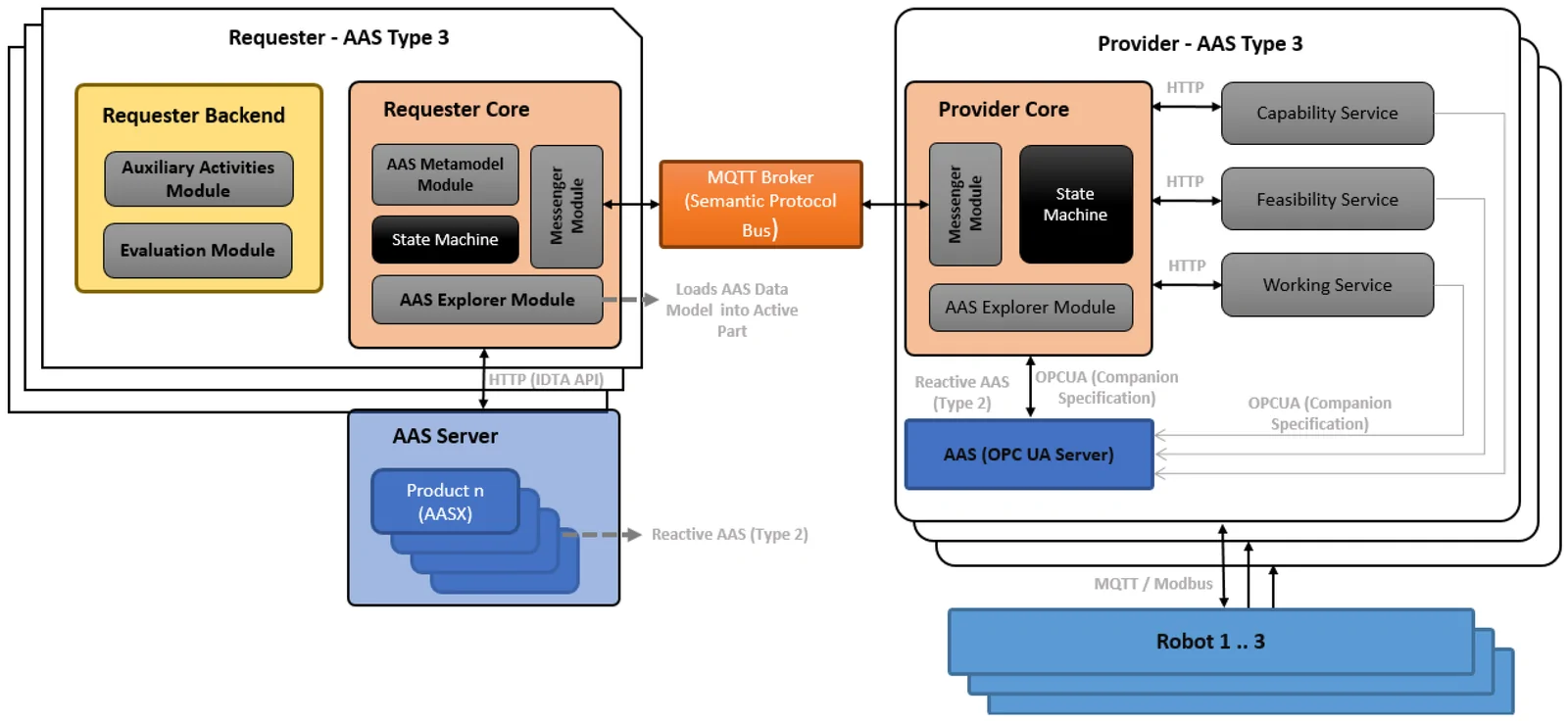
Overall architecture used to validate requester–provider negotiation, asynchronous messaging, and the interaction between active and reactive AAS layers.

**Figure 5:**
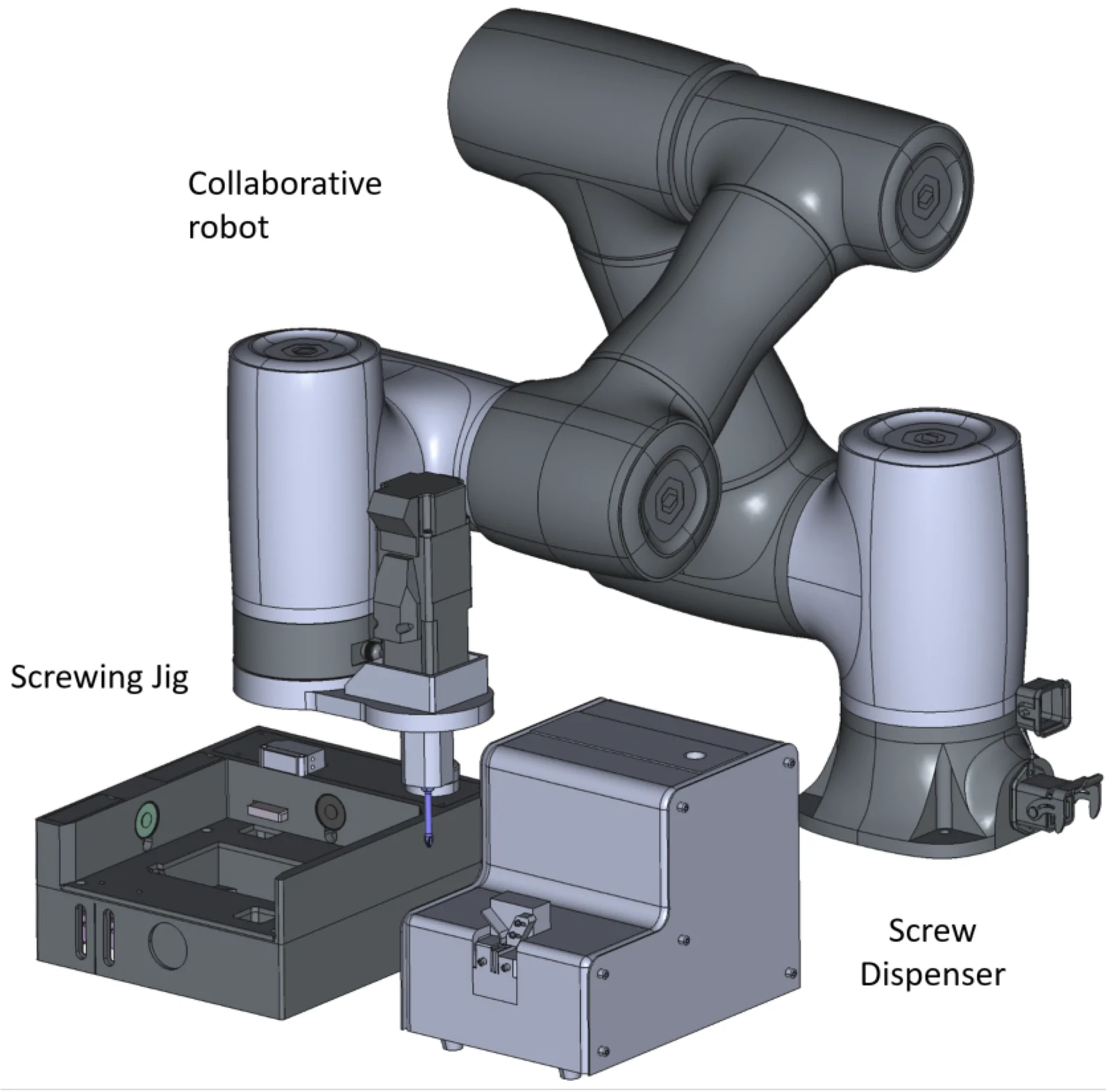
Provider asset detail: the six-axis collaborative robot carries the screwdriving end-effector over the fixture, while screws are fed by the dispenser. The robot is a CR3 collaborative arm (Dobot CR3, Shenzhen Yuejiang Technology Co., Ltd., Shenzhen, China; supplied in Brazil by Minipa Robotics), with 3 kg rated payload and 620 mm reach.

Validation was conducted on a robotic screwdriving demonstrator built at the Universidade do Estado do Amazonas. The demonstrator reproduces a finished-product assembly station of the type deployed in the Manaus industrial district and is built to industrial standards, but it does not operate as part of a live production line. It comprises three six-axis collaborative robots with screwdriver tooling, an input jig, three screwing jigs, an output jig, screw dispensers, and a linear transport system that moves products between stations (Figure 3). Product variants are requester AASs; the robots and auxiliary devices are provider AASs. Reactive submodels capture execution capabilities (torque ranges, axis limits, safety envelopes) and interface endpoints (HTTP, MQTT, OPC UA). The active layer runs as microservices that execute bidding and orchestration using the *ActiveSkills* and *ActiveSteps* definitions for each variant, with no structural change to the reactive layer. Figure 4 summarizes the end-to-end flow exercised in this demonstrator, while Figure 5 provides a detailed view of a provider-side robotic asset.

### 2.7. Products as Type-3 AAS with BaSyx Support

Product variants are modeled as type-2 AASs hosted on an Eclipse BaSyx stack, exposed via HTTP through the BaSyx services. When the corresponding active part is instantiated, these products become type-3 AASs. BaSyx offers registry, submodel server, and proxy components, so products can be created in the server by manufacturing management systems through the standardized IDTA API [20,21]. Each product AAS exposes execution-neutral submodels (bill of process, quality constraints) plus *ActiveSteps* that map variant-specific sequences. BaSyx handles discovery and access control while the active layer consumes the *ActiveSteps* submodels to drive negotiation and orchestration. This separation keeps the product definitions reusable and lets new variants be onboarded by deploying only active-layer packages, without changes to BaSyx-managed reactive assets.

### 2.8. Requester and Provider Views

**Figure 6:**
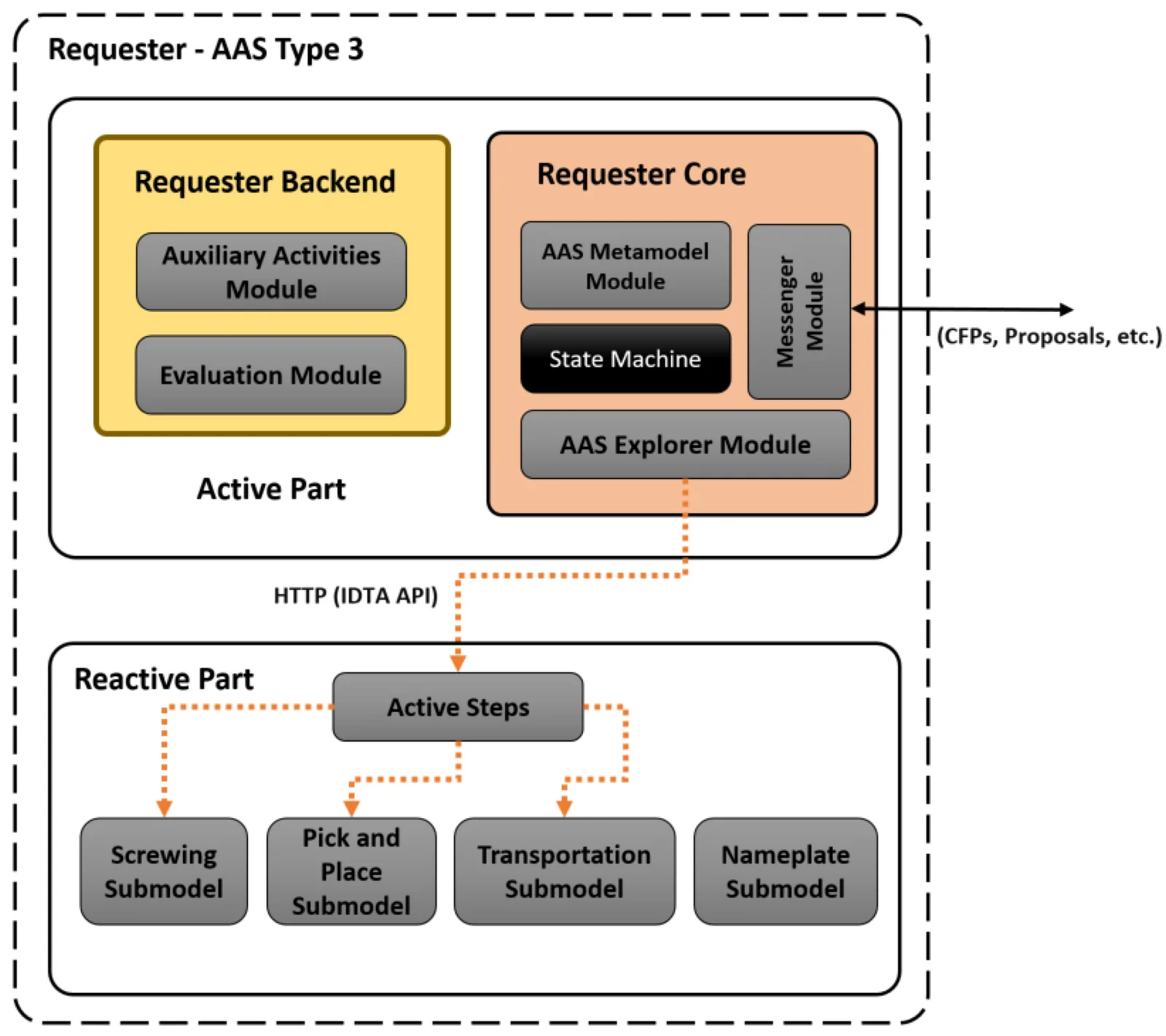
Requester AAS view highlighting *ActiveSteps* and negotiation interfaces.

**Figure 7:**
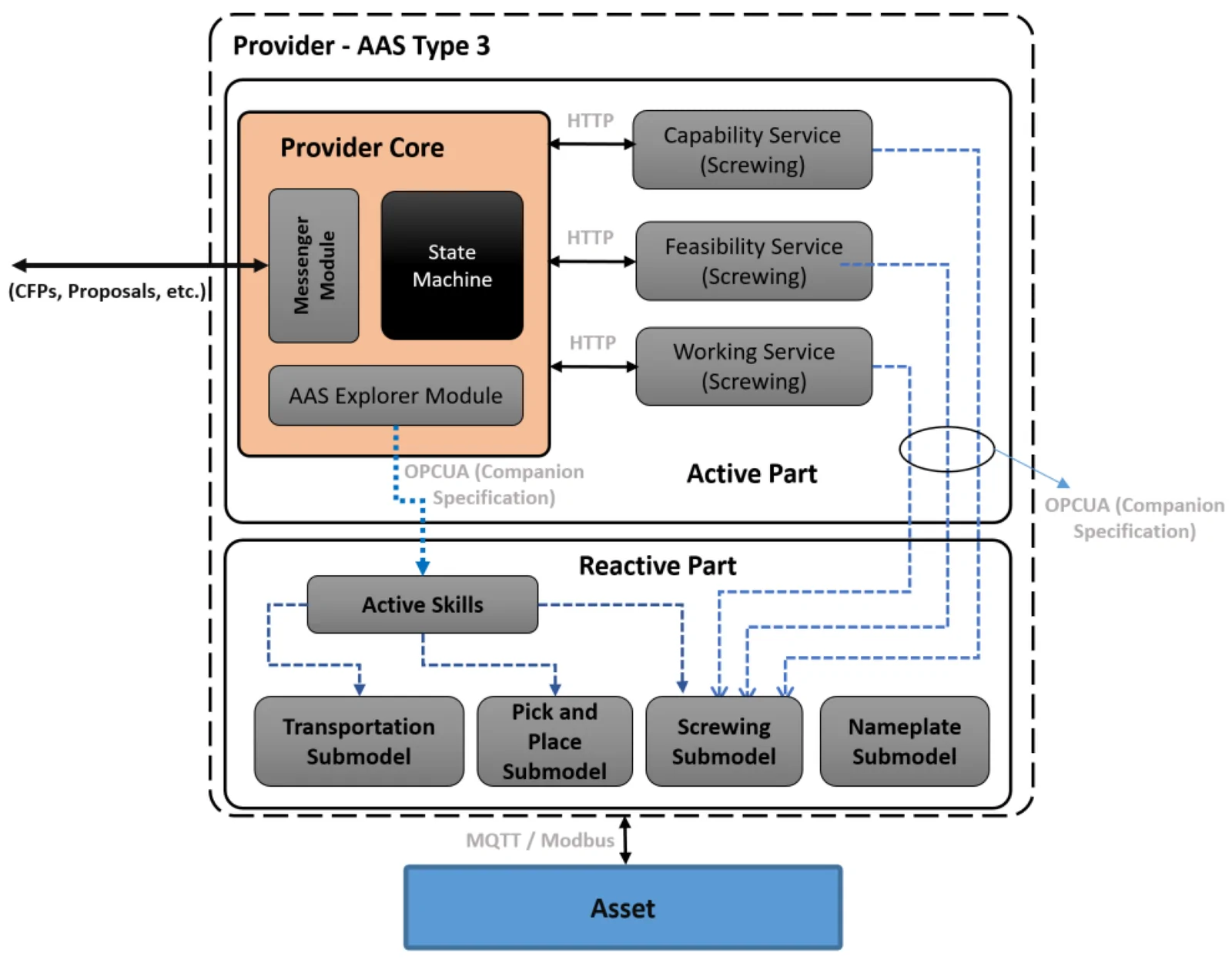
Provider AAS view highlighting *ActiveSkills* services and execution submodels.

To make requester and provider roles explicit, Figure 6 shows the requester AAS with its *ActiveSteps* and negotiation endpoints, while Figure 7 shows the provider AAS exposing capability and feasibility services alongside its execution submodels.

The experimental protocol assessed two complementary aspects. The first was architectural validation of the end-to-end negotiation flow. The second was quantitative validation of the AI-assisted capability-checking service embedded in the provider decision chain.

### 2.9. Model Selection Protocol

The decision core was not fixed a priori. It was selected through an iterative protocol in which each candidate configuration, a combination of prompt strategy and language model, was executed over a versioned test suite and characterised by two metrics, reported separately. Structural conformity, CE, is the fraction of inferences that returned a schema-conformant JSON verdict, and measures whether the output can be consumed by the provider state machine at all. The F1 score over the capable/not-capable decision measures classification quality. F1 was preferred over accuracy because the early suites were unbalanced, and because false positives and false negatives carry different operational consequences in a production cell. Table 2 summarises the trajectory.

Three findings drove the selection. Prompt structure alone did not resolve the task: moving from direct pattern matching to a five-part structured prompt (role, context, matching rules, task and response schema) raised accuracy but lowered F1, so the model was answering more conservatively rather than more correctly. Supplying the model with deterministic tools for literal comparison, interval containment, and numeric ordering degraded every metric, because tool invocation introduced failure modes (malformed tool arguments and loss of the required response schema) that outweighed the arithmetic it removed. Only the migration to models with embedded reasoning brought the task into a usable range.

The final configuration was chosen on operational rather than statistical grounds. GPT-5 reached the required decision quality but averaged 24 s per evaluation and occasionally exceeded 5 min, which is incompatible with production-time coordination, and it is reachable only through a proprietary API. GPT-OSS-20B is an open-weight model that can be hosted inside the plant boundary, which also avoids transmitting capability declarations (process know-how such as torque envelopes and cycle times) to a third-party service. It matched the decision quality of GPT-5 on the same suite at a response time compatible with asynchronous negotiation.

Two limitations of this protocol are stated explicitly. The test suite evolved across the trajectory as failure modes were identified, so the rows of Table 2 are not mutually controlled comparisons; they document a sequence of engineering decisions rather than a single factorial experiment. From the migration to reasoning models onwards, the generator seed was deliberately released rather than fixed, so that repeated executions of the same case expose the variability of the model instead of masking it. Each case is therefore executed several times, and decision quality is measured as the proportion of correct verdicts across repetitions.

### 2.10. Capability-Checking Evaluation Protocol

The capability-checking service used the GPT-OSS-20B open-weight model to analyze JSON-based submodels and determine whether a provider met the requirements issued by a requester. The evaluation focused on semantic matching and constraint verification, including range-based checks over operational parameters. This design is aligned with recent work on semantic capability inference, semantic service composition, and LLM-assisted AAS information modeling in Industry 4.0 environments [13,22,23,24]. Following the protocol of Section 2.9, inference was not forced to be deterministic: the generator seed was left free and each test case was executed with five repetitions, so that the reported decision quality reflects the variability of the model under repeated invocation.

Two campaigns are reported, and they answer different questions. The *timing and cost* campaign characterises the service on three infrastructures and reuses the suite that closed the selection trajectory of Section 2.9: 145 unique cases, five repetitions on the serverless infrastructure, giving 725 evaluations, and one repetition on each rented GPU. The *decision-quality* campaign, reported in Section 3.3, uses a larger suite built specifically to test the algorithm rather than to select a model: 454 unique cases, balanced between compatible and incompatible, each executed five times, giving 2270 evaluations. Both campaigns exercise the *Screwing* activity with the same internally developed submodel and the same system prompt.

Reference labels are established by construction and not by annotation. Each case is built by instantiating a requester requirement together with a provider capability declaration whose compatibility follows from the design of the case, so the expected verdict is fixed before the model is invoked. No expert assessment was applied after the fact, and no deterministic reference implementation was used to generate the labels; a rule-based checker would resolve the numeric-bound cases but not the ones that turn on semantic interpretation of heterogeneous submodel structures. Because each case is executed five times, the reported confusion matrices count executions rather than independent samples. Unique cases and executions are reported separately throughout, since the repetitions are not independent samples.

The 454 cases are organized in four groups, one per failure mode the algorithm has to survive, and each group is balanced between positive and negative cases (Table 3). *Semantic incompatibility* passes submodels that are wholly or partly disjoint from the capability requested, for instance a *PickAndPlace* declaration offered against a *Screwing* request. *Insufficient inferential evidence* covers requirements the provider declaration does not carry enough data to settle. *Limiting properties* varies numeric intervals and values so that the request falls outside what the declaration supports, such as a torque the maximum of the offered capability does not cover. *Invalid or inconsistent data* mixes data types, passing integers where floating point is expected, hexadecimal values as integers, and inverted numeric intervals. Balancing each group is what makes accuracy interpretable alongside F1: a classifier that always answered “not capable” scores 50% on this suite.

Every case pairs a provider capability declaration with a requester requirement, spans the operational parameters of the submodel including torque set point and range, screw dimensions and fixture constraints, and uses the same system prompt and model configuration described in Section 2.9. Model verdicts were scored against the capable/not-capable label fixed when the case was built. Outputs that were not schema-conformant JSON were counted as structural failures through the CE metric and excluded from the F1 computation, so that classification quality was measured only over verdicts the provider state machine could consume. For the comparative timing analysis on dedicated GPU VPSs rented through Vast.ai, the 145 cases of the timing campaign were executed once on each infrastructure, an RTX 5090 configuration and an RTX PRO 6000 WS configuration. These experiments refer to the semantic capability-checking component, not to complete bidding-process executions. The measured indicators included response time, token throughput, and operational cost per capability evaluation, with cost analysis reported for the Groq API execution. Decision quality was analyzed using confusion-matrix logic, with particular emphasis on minimizing false positives, since such errors could lead a provider to accept a task for which it lacks physical capability.

The decision-quality campaign was also used to compare five open-weight reasoning models, two of the GPT-OSS family and three of the Qwen3.5 family, so that the reported behavior is not a property of a single model. All five were executed over the same 454 cases with the same five repetitions.

### 2.11. Instrumentation and Metric Computation

The demonstrator was instrumented at the message, deployment, service, and container levels. Negotiation messages were persisted from the MQTT topics used by the requester–provider protocol, including CFPs, provider responses, requester acceptances, requester rejections, and service-completion messages. RFID events emitted when a product entered the active-layer deployment flow were stored as deployment-start markers. Service-level timing events were published by requester and provider components, while Docker resource samples were periodically collected from the active-layer containers. All timestamps were normalized to UTC before metric computation.

The CFP response time $t_{cfp}$ measures the requester-side elapsed time between sending a call for proposal and receiving the first valid provider proposal. For each CFP message $m_{cfp}$ with UTC-normalized timestamp $\tau (m_{cfp})$, the dashboard identifies provider messages of type proposal whose in_reply_to field references $m_{cfp}$, excluding refuse and not_understood responses. If $P(m_{cfp})$ is the set of such proposals, the per-CFP latency is computed as

(1) $$t_{cfp}\left(m_{cfp}\right)=\min_{p\in P(m_{cfp})}\tau \left(p\right)-\tau \left(m_{cfp}\right)$$

Here, τ(x) denotes the UTC-normalized timestamp of message *x*. The reported values are the median and 95th percentile over all CFPs in the automated measurement campaign for which at least one proposal was observed.

The active-layer deploy time $t_{deploy}$ is inferred from the product-deployment trace. A deployment attempt starts when the spawner-side RFID event for a product asset is recorded. The deployment is considered successful when the newly activated requester emits its first CFP before the next RFID deployment event for the same product. For a product asset *a*, with RFID timestamp $\tau_{rfid}\left(a\right)$ and first CFP timestamp $\tau_{cfp}^{first}\left(a\right)$ observed before the next RFID event for *a*, the deploy time is computed as

(2) $$t_{deploy}\left(a\right)=\tau_{cfp}^{first}\left(a\right)-\tau_{rfid}\left(a\right)$$

This metric therefore captures the elapsed time from product introduction into the active-layer deployment flow until the product becomes negotiation-capable. It is an operational proxy for active-layer activation, not a low-level container-health probe.

The CFP throughput $\lambda_{cfp}$ is computed over the automatic-simulation interval rather than over the full dashboard time horizon. The interval starts at the first CFP emitted by requester A0000007, which marks the beginning of the automated product sequence, and ends at the last CFP emitted by a requester in the automatic-sequence identifier range. If $N_{cfp}$ CFPs are observed between $\tau_{start}$ and $\tau_{end}$, throughput is computed as

(3) $$\lambda_{cfp}=\frac{N_{cfp}}{(\tau_{end}-\tau_{start})/60}$$

Using this interval prevents older manual tests or isolated CFPs from diluting the throughput estimate of the automated run.

The deploy success rate $S_{deploy}$ is computed from the same RFID-to-CFP linkage used for $t_{deploy}$. A deployment attempt is counted for every product RFID event. It is counted as successful when a first CFP from the same product requester is observed before the next deployment event for that product. Thus,

(4) $$S_{deploy}=\frac{N_{deploy}^{success}}{N_{deploy}^{attempts}}$$

This definition measures successful activation as observed by the negotiation layer. Manual rollbacks or container-level failures would require explicit spawner status events to be measured separately.

The allocation baseline was computed offline to isolate the effect of provider selection. The bidding-based allocation uses the observed winning provider for each completed working-service execution. The equal-share baseline reassigns the same number of completed jobs to the three robots in round-robin order. Each reassigned job is estimated using the empirical mean working-service time of the robot receiving that job. For robot *r*, with observed working-service durations $D_{r}$, the baseline service time assigned to *r* is ${\bar{d}}_{r}=\mathrm{mean}\left(D_{r}\right)$. For an allocation policy π, the estimated parallel makespan is the maximum accumulated assigned workload among the three robots:

(5) $$T_{makespan}^{\pi }=\max_{r}\sum_{j\in A_{r}^{\pi }}d_{j,r}^{\pi }$$

where $A_{r}^{\pi }$ is the set of jobs assigned to robot *r* under policy π. For the observed bidding allocation, $d_{j,r}^{\pi }$ is the measured working-service duration of job *j*. For the equal-share baseline, $d_{j,r}^{\pi }={\bar{d}}_{r}$ for each job reassigned to robot *r*. Throughput is then computed as the number of completed jobs divided by the estimated makespan in hours. This baseline should be interpreted as an allocation-policy comparison; it does not re-simulate transport contention, queueing, or cell-level disturbances.

## 3. Results

### 3.1. Architectural Validation

The proposed architecture sustained a stable end-to-end bidding flow in the demonstrator. The requester emitted CFPs, providers evaluated technical and operational constraints, proposals were returned asynchronously, and the selected provider executed the contracted service while preserving communication feedback. This result is relevant because it demonstrates that proactive digital twin behavior can be obtained without embedding all decision logic directly inside a monolithic AAS server.

The three properties defined in Section 2.1 were observed as follows. Upgrading a demonstrator asset from type-2 to type-3 inserted two submodels: on the requester side the *ActiveSteps* submodel and the submodel declaring the capability required by the product; on the provider side the *ActiveSkills* submodel and the submodel declaring the capability offered by the resource. No submodel already present was refactored. The upgrade cost one restart of the reactive part of each provider, while on the requester side the submodels were inserted through the BaSyx API with the server running, so no restart was needed there. Subsequent active-layer rollouts inserted no submodel, changed no submodel structure, and required no restart of the reactive layer, so consumers reading the reactive AAS were not interrupted by them. The element values written back by the active layer during operation, such as the step status of *ActiveSteps*, remained readable through the canonical AAS interfaces throughout.

The active-layer bundle deployed for each provider comprised one generic provider-core image, instantiated once per robot, together with the capability, feasibility and working services of the screwing scenario. The core resolved the reference held in its own *ActiveSkills* submodel to reach the capability submodel of its resource, so no core variant per robot was built; what varies across scenarios is confined to the services the core calls. Working services were updated with no restart of the reactive layer.

The separation between the reactive AAS and the active layer also let the reactive side be exposed by pure OPC UA resources or by AAS-oriented stacks with no change to the requester logic. This was observed for the resources of this demonstrator and is not a general interoperability result. It does suggest that brownfield environments, in which computational capacity, connectivity constraints and deployment technology vary across assets, are within reach of one requester implementation.

### 3.2. Response Time and Cost of the Capability Check

This subsection reports the timing campaign of Section 2.10: the 145-case suite executed on three infrastructures. It characterizes how fast and how cheaply the service runs, not how well it decides; decision quality is reported in Section 3.3. On the serverless infrastructure, the mean time per evaluation was 1.73 s over 725 executions, with a generation throughput of 893 output tokens per second as reported by the provider API.

The two rented GPU configurations were procured to characterize self-hosted execution, and their timings carry a measurement caveat that has to be stated before they are read. Both machines were located on a different continent from the client, so the client-observed means of 5.54 s and 4.83 s are dominated by network transit and are not comparable with the serverless figure. For the RTX 5090, server-side logs place the mean at 2.30 s once transit is excluded, and give a generation throughput of 640 output tokens per second. For the RTX PRO 6000 WS, the corresponding logs were not captured, so no server-side time and no throughput are reported for that configuration. Table 4 states which figures are client-observed and which are server-side, and marks the quantities that were not recorded.

None of these differences affects the conclusion drawn from the measurement. The capability check runs inside a negotiation whose end-to-end cycle takes 44 to 82 s, so the decision service is not the limiting element on any of the three infrastructures.

The Groq experiments were priced with the published rates for GPT-OSS-20B on that service: USD 0.075 per million input tokens, USD 0.037 per million input tokens served from cache, and USD 0.30 per million output tokens. At list price, one evaluation of 6924 input and 770 output tokens costs

(6) $$C=6924\times 0.075\times 10^{-6}+770\times 0.30\times 10^{-6}=7.50\times 10^{-4}\;\mathrm{USD}$$

so a full round of 725 evaluations was budgeted at USD 0.544. The round actually was billed at USD 0.51, because part of the input context was served from the cache at the reduced rate. The measured cost per evaluation is therefore USD $0.51/725=7.0\times 10^{-4}$. This suggests that LLM-assisted capability checking may already be economically viable for asynchronous decision steps in manufacturing systems, provided that strict real-time constraints are not imposed.

### 3.3. Balanced Validation of the Capability Check

The decision-quality campaign of Section 2.10 evaluates the deployed configuration over the 454 balanced cases of Table 3, each executed five times, for 2270 evaluations. Every one of the five models returned a schema-conformant JSON verdict in every execution, so structural conformity was 100% throughout and is not tabulated per model.

**Figure 8:**
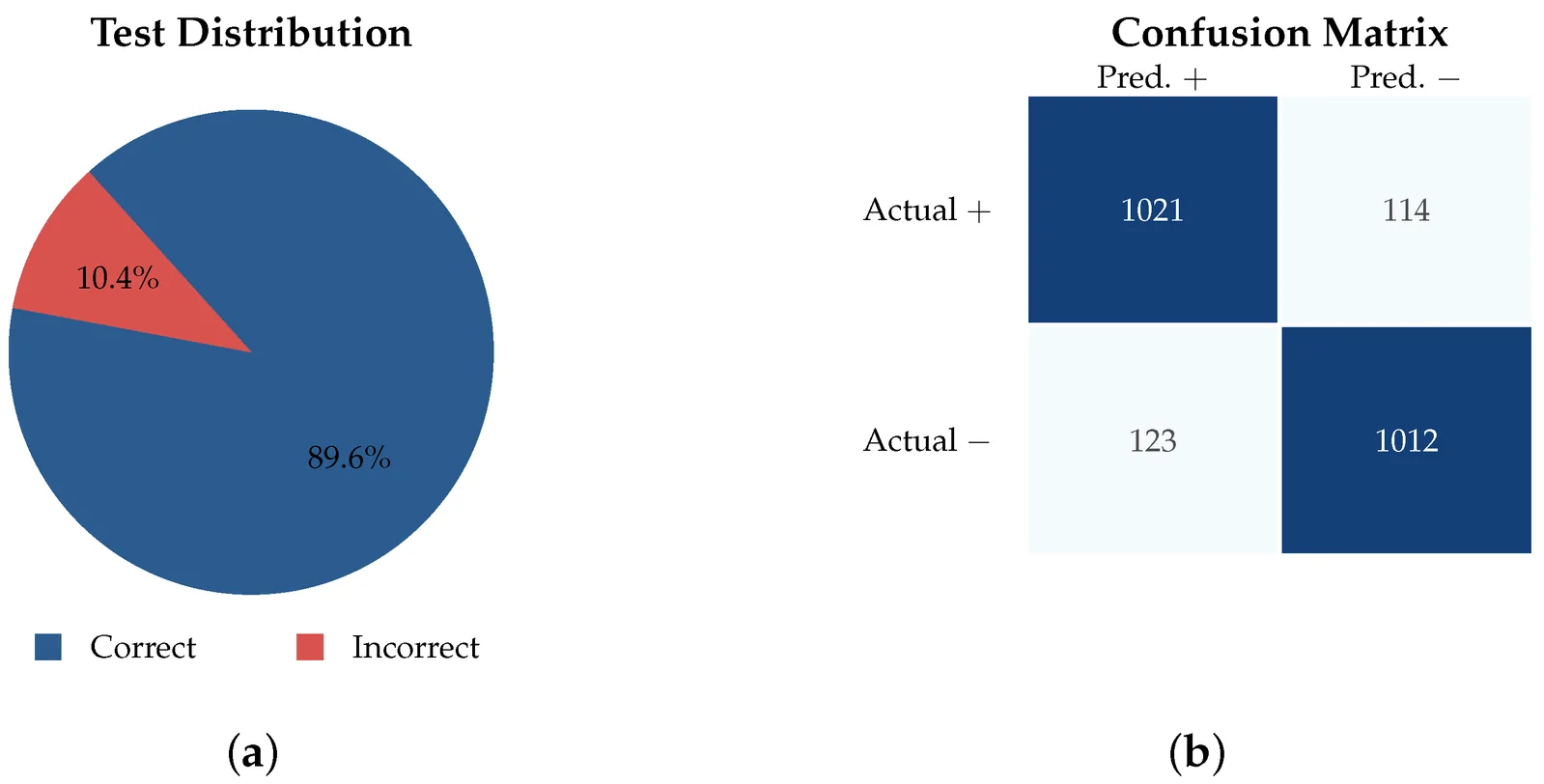
Capability-checking results for GPT-OSS-20B over the 454 balanced cases, 2270 executions. (**a**) Share of executions whose verdict matched the label fixed at construction (89.6% correct, 10.4% incorrect); (**b**) resulting confusion matrix, with *capable* as the positive class.

For GPT-OSS-20B, the model the demonstrator runs, 2033 of the 2270 executions returned the label fixed when the case was built, an accuracy of 89.56%. Because the suite is balanced, accuracy is interpretable here and agrees with F1, which is 89.60%. Figure 8 gives the distribution and the confusion matrix. Precision is 1021/1144=89.25% and recall is 1021/1135=89.96%. The false-positive rate is 123/1135=10.8%, and it is the figure that matters operationally, since a false positive commits a provider to a task beyond its physical capability. These quantities are computed over executions and not over independent cases: the 2270 executions are 454 unique cases repeated five times each, so they characterize the behavior of the service under repetition and not the variance of an independent sample.

This suite is harder than the one that closed the selection trajectory, and the difference should be read deliberately. On the 145-case selection suite, the same model reached 96.70% accuracy and 92.00% F1 (Table 2), with a false-positive rate of 2.1%. That suite was unbalanced, with 21% positive cases, and it was assembled to discriminate between candidate configurations. The 454-case suite was assembled to find the limits of the selected one, and it adds cases whose verdict turns on evidence the provider declaration does not carry. The lower figures are the ones that should be quoted for the algorithm.

Table 5 reports the five models over the same suite, aggregated and by failure-mode group. Three readings follow from it. First, model size does not order the results: GPT-OSS-120B leads on three of the four groups yet loses the aggregate to GPT-OSS-20B, because it is 3.2 points weaker on invalid and inconsistent data, the largest group. Second, the length of the reasoning trace is inversely related to accuracy across the range observed, from 544 tokens at 89.56% to 3599 tokens at 70.66%, so a longer deliberation is not evidence of a better verdict and the reasoning budget is a cost lever rather than a quality lever. Third, and most consequential for deployment, *insufficient inferential evidence* (G2) defeats every model tested: none exceeds 74.73% on that group, while three of the five sit above 98% on semantic incompatibility. The failure mode is therefore not semantic matching, which the models handle well, but knowing when a declaration does not carry enough information to decide at all. Missing evidence is exactly the case in which a conservative refusal is correct and an optimistic verdict becomes a false positive, which is where the 10.8% of Figure 8 originates.

Two limits of this campaign should be stated. The five models are open-weight reasoning models of two families, so the comparison does not speak to proprietary models, and GPT-5 was not re-run on this suite because it was excluded on response time and hosting grounds in Section 2.9. And the cases were written by hand, which bounds how large the suite can grow and leaves open whether the four groups cover the failure modes of activities other than screwing.

### 3.4. Token Consumption and Cost Structure

The token-consumption analysis separates where the tokens are spent from where the cost is incurred, because input and output are not priced alike. Input dominates the token budget: the provider submodel, the requester submodel and the system prompt account for 90.0% of the tokens processed per evaluation. Cost follows a different distribution. Output is priced four times higher than input, so the 10.0% of tokens that the model generates carry 30.8% of the cost, and the reasoning trace alone accounts for 25.3%, second only to the provider submodel. Two levers therefore act on economic viability, and they are independent: payload compaction and submodel pruning reduce the input side, and control of the reasoning effort reduces the output side. Table 6 summarizes token distribution and corresponding cost shares per execution.

### 3.5. Measured Evaluation Metrics for Mass-Customized Production

The demonstrator dashboard persists MQTT negotiation messages, RFID events emitted when products are introduced into the active layer, service-level timing events, and periodic Docker resource samples. Table 7 summarizes the operational metrics obtained during the automated demonstrator measurement campaign. All counts and latencies reported in this section are computed over the automatic-simulation interval, which starts at the first CFP emitted by requester A0000007 and ends at the last CFP of the automatic sequence. The dashboard also holds earlier manual tests, which this window excludes, so totals read directly from the raw event log are larger than the ones reported here.

**Figure 9:**
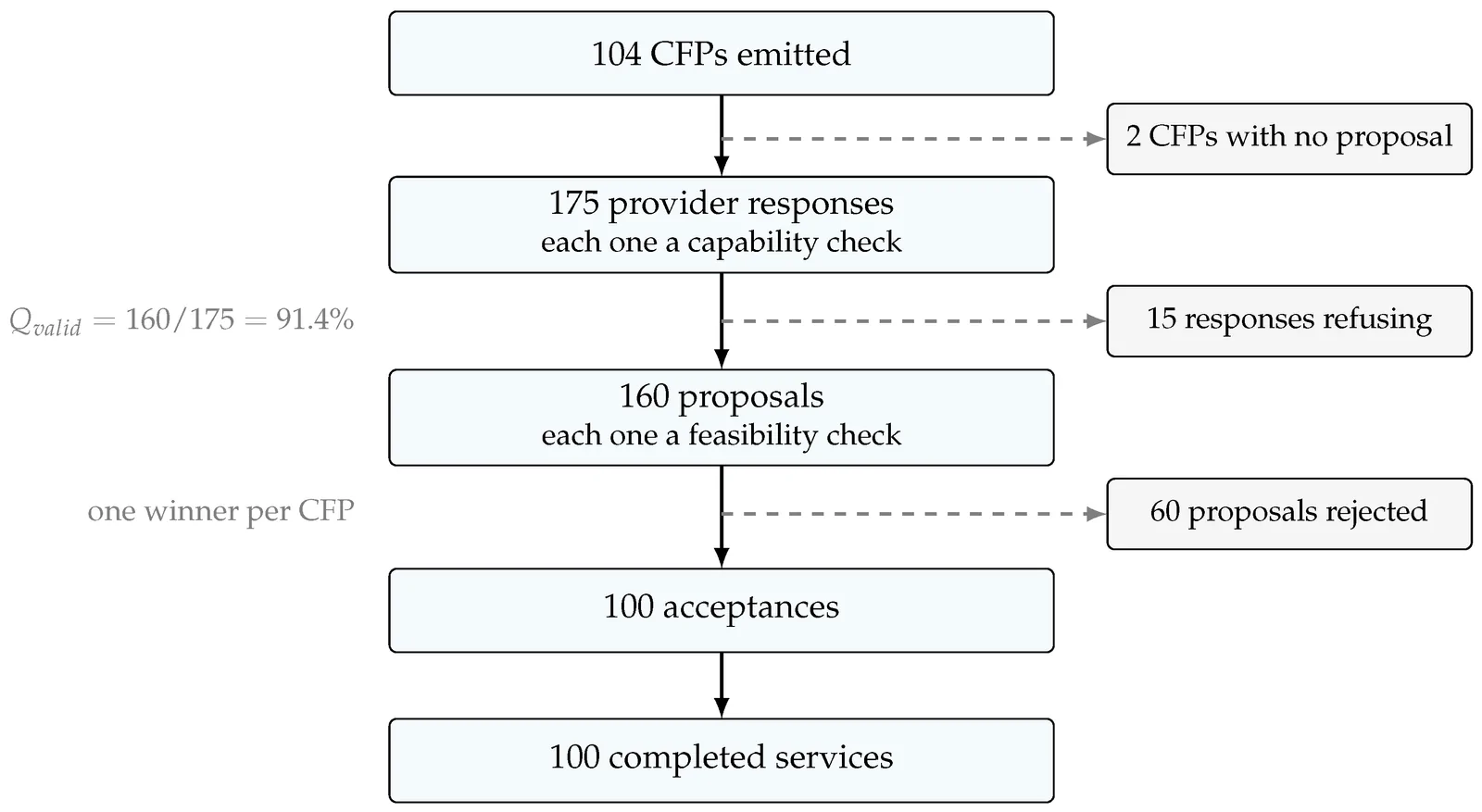
Relationship between the sample sizes reported in Table 7, Table 8 and Table 9. Each stage of the negotiation produces a different count, and the dashed branches account for the differences.

The metrics in Table 7, Table 8 and Table 9 are drawn from different stages of the same negotiations, which is why their sample sizes differ. Figure 9 traces the stages and the counts.

Two of the counts deserve an explicit note. The 175 provider responses average 1.68 per CFP rather than the three that the cell’s three robots might suggest, because a provider executing a contracted service does not bid. A CFP issued while all three robots are free draws three proposals; the next CFP, issued while the winner is still working, draws two; the one after that draws one. Responses per CFP therefore fall as robots become occupied and recover as they complete, and 1.68 is the average over a campaign in which the product sequence kept the cell close to saturation. The 60 rejected proposals are the losing bids of CFPs that received more than one, since each CFP awards a single provider.

The resource metrics were obtained by sampling the Docker statistics of the active-layer containers at a fixed period and attributing each sample to the CFP window it falls in. Two consequences follow, and both are stated in Table 8 instead of being smoothed over. First, a CFP whose window is shorter than the sampling period contains no sample at all, which is why the CPU and memory figures rest on 83 and 87 of the 104 windows. Second, the sample is a point reading rather than an integral, so the maxima report the highest instantaneous value observed and not a peak guaranteed to have been captured.

Network usage per CFP was collected by the same mechanism and is not reported. The per-container counter deltas over CFP windows rounded to zero at the resolution recorded, which cannot be right: the negotiation itself is carried over MQTT within those windows, so the traffic exists by construction. The measurement did not isolate the interfaces that actually carry the negotiation, and reporting the value would have described the instrument and not the system. Quantifying negotiation traffic from message counts and payload sizes is left to a dedicated measurement.

The sample sizes reported across Table 7, Table 8 and Table 9 differ because they correspond to successive stages of the same negotiation funnel, and are not expected to coincide. Each CFP that received at least one proposal (102) is answered by one or more providers, and each responding provider runs one capability check, so the 175 capability checks exceed the number of CFPs. Of the 175 provider responses, 160 were valid proposals, which matches the 91.4% valid-response rate ($Q_{valid}$) in Table 8 and fixes the 160 feasibility checks. Downstream, 100 proposals were accepted and 100 working services completed, which sets the sample size of the acceptance, evaluation, and execution metrics. The capability-checking campaigns belong to a separate offline evaluation that characterizes that service in isolation and are not part of the live demonstrator counts: 145 unique cases and 725 evaluations for the timing and cost figures of Table 4 and Table 6, and 454 unique cases and 2270 evaluations for the decision-quality figures of Table 5.

Table 9 reports the internal timing events published by the active services during the measurement campaign. These values complement the end-to-end CFP metrics by separating requester-side evaluation, provider-side capability and feasibility checks, and execution time.

To contextualize the provider-selection behavior, an offline equal-share baseline was computed from the same 100 completed working-service executions, as summarized in Table 10. The baseline assigns jobs to the three robots in round-robin order and estimates each assigned job using the empirical mean service time of the assigned robot. This comparison isolates the allocation policy: the proposed bidding mechanism naturally concentrated more jobs on the fastest robot, while the equal-share baseline forced an approximately even distribution. This is an allocation-policy estimate, not a full cell simulation, since it does not re-simulate transport contention, queueing, or cell-level disturbances. The resulting 38% relative throughput gain should therefore be read as a preliminary, demonstrator-level indication of the effect of bidding-based selection, not as a demonstrated production throughput improvement.

The remaining planned metrics, including storage per active package ($STO_{pkg}$) and onboarding lead time from active-step definition to first successful execution ($t_{onboard}$), require additional instrumentation beyond the data emitted during the campaign.

### 3.6. Assessment of the Hypotheses

**H1** is supported for the requester side. Product variants were held as modeling files in the BaSyx server and their active part was instantiated on demand: 100 of 100 deployment attempts reached the negotiation layer, at a median activation time of 8 s (Table 7). Upgrading a demonstrator asset inserted two submodels and refactored none of those already present, and subsequent rollouts introduced no structural change. The evidence covers 100 product variants in one cell and does not address how activation time behaves as the number of concurrent variants grows.

**H2** is partially supported. One provider-core image, instantiated once per robot, drove the three providers of the cell, with the capability, feasibility and working services of the screwing scenario deployed alongside it, and working services were updated with no restart of the reactive layer. Two limits should be read with that result. The reuse demonstrated is of the core and not of the whole active layer, since a new scenario still calls for its own domain services. And the claim that this agnosticism makes instantiation automatable follows from the design and not from a measurement, since the tool that would generate the active part from the submodels is under construction and outside the evaluation scope of this paper.

**H3** is supported within the evaluated envelope. Negotiation sustained a median of 4 s from the call for proposal to the first proposal and 44 s to a completed service cycle (Table 7), and the capability-checking service returned a schema-conformant verdict in every execution, with an F1 of 89.6% over the balanced suite. These figures characterize asynchronous coordination in one cell at the observed load. They do not extend to hard real-time control, for which the reported latencies are orders of magnitude too large.

## 4. Discussion

The results show a proactive digital twin operating as a decision-capable entity and not only as a richer data container. Previous work has discussed the relationship between AAS and digital twins [5,6], explored proactive AAS concepts [7,8,9,10], and proposed semantic or agent-based mechanisms [13,14,15]. This study adds a modular deployable implementation for mass customization that separates the active and reactive layers explicitly and exercises that separation on a demonstrator built to industrial standards.

Table 11 positions the proposal against the families of related work along the dimensions most relevant to mass-customized deployment. The entries reflect the aspect each cited work reports as its primary focus and are not an exhaustive capability audit of those systems.

Proactive and type-3 behavior is, by itself, not new: order-driven proactive AAS [7,8], multi-agent realizations of type-3 [9,10,14], and containerized proactive AAS [19] all provide it, and capability–skill–service work supplies the semantic matching layer [11,12,13]. Separating decision logic from asset data is, on its own, ordinary service-oriented design and no contribution is claimed for it. What is specific to this work is the combination of three properties that are usually addressed in isolation. The type-2 to type-3 upgrade is *additive*: existing submodels keep their structure and their consumers, and the upgrade inserts only the pair of contract submodels and the services that read them, at the cost of one restart of the reactive part. From then on the active layer is redeployed, replaced, or rolled back with no restart of the reactive layer, so consumers of the reactive AAS are not interrupted by changes to decision logic. And the boundary is expressed as an explicit submodel-level contract, which makes it declarative instead of an ad hoc integration. The distinction is therefore not that data and logic are separated, but that the separation is declared in the AAS metamodel itself and is exercised as a deploy-time lifecycle boundary on industrial-grade hardware.

One dimension of the table would not favor the proposal. The *ActiveSteps* and *ActiveSkills* submodels are defined for this architecture and are not IDTA-standardized (Section 2.4), so the contract is portable within an implementation and not yet across vendors. Standardizing the two templates is the condition for that, and Section 5 states it as future work.

The architectural contribution is not merely the addition of external microservices around an AAS. Its central claim is the definition of a stable boundary between the reactive shell, which preserves standardized asset representation and legacy interoperability, and the active layer, which can be deployed, replaced, and replicated according to product-variant demand. The *ActiveSkills* and *ActiveSteps* submodels make this boundary explicit by defining which decision and orchestration functions may evolve independently while still referencing the certified execution-oriented submodels. This separation turns proactive behavior into a lifecycle-managed architectural layer rather than an ad hoc integration pattern.

From a systems perspective, the main advantage is preserving the reactive digitalization while rolling out new active logic. Assets that already expose data through a reactive shell can participate in proactive orchestration without rework. This is valuable in mixed environments that combine legacy equipment, emerging AAS stacks, and resource-constrained field devices. The architecture also aligns with interoperability initiatives that combine AAS with Gaia-X, OPC UA, Manufacturing-X, and capability-centered workflows [16,17,18,25].

The quantitative results also position the capability-checking service within a realistic operating envelope. Latency in the order of seconds is not suitable for hard real-time control loops, but it is acceptable for asynchronous negotiation, dispatching, and planning tasks. Therefore, the architecture is better interpreted as a coordination-level digital twin framework rather than a replacement for low-level deterministic control.

An additional practical indication of relevance is that the same architectural principles are currently being reused in two ongoing R&D initiatives at the TPV Manaus R&D center. One initiative applies intelligent digital twins to warehouse inventory operations with drones and warehouse assets represented as proactive twins. A second initiative consolidates the modular architecture into AASistant, a platform intended to model, instantiate, deploy, and monitor type-1, type-2, and type-3 digital twins for smart Industry 4.0 components, and which already implements the automated active-layer deployment pipeline used in this study. Although these initiatives are outside the formal evaluation scope of the present paper, they suggest that the proposed architecture is not limited to a single demonstrator and can serve as a reusable foundation for broader cyber-physical infrastructures.

The study has limitations, and two of them concern comparisons that were not made. A standalone deterministic validator was not run as a baseline. The closest experiment reported here supplied the decision core with deterministic tools for literal comparison, interval containment and numeric ordering, and it degraded every metric (Table 2), because tool invocation introduced failure modes that outweighed the arithmetic it removed. That result compares a model with and without deterministic assistance, which is not the same as comparing the model against a rule-based checker. Such a checker would settle the cases that turn on numeric bounds and would not settle the ones that turn on semantic interpretation of heterogeneous submodel structures, which are the cases the service exists to handle. A hybrid pipeline that routes each case to the mechanism suited to it is the natural next step, and Table 5 now indicates where the split lies: semantic incompatibility and limiting properties are resolved above 97% by the deployed model, while the cases that turn on insufficient evidence stay below 75% for every model tested, so those are the ones a deterministic guard or an explicit abstention would have to absorb.

The architecture was also not compared against a monolithic implementation. That comparison calls for building and instrumenting a second complete system on the same cell, which is beyond the scope of a feasibility study. Its design is nonetheless defined: the same product variants and the same CFP sequence executed against an AAS server with the decision logic embedded, comparing the time to introduce a variant, the interruption to reactive-layer consumers during an update of decision logic, and the negotiation latency.

The validation took place in a single cell and not in a multi-line plant. The false-positive rate of 10.8% measured on the balanced suite (Section 3.3) is not acceptable on its own for a step that commits a resource to a task, and it sets a condition rather than a caveat: a deployment must either place the decision behind a deterministic guard on the numeric bounds, or treat a capable verdict as a proposal that execution can still refuse, which is how the demonstrator uses it. Results should be read as evidence of feasibility for coordination-level reasoning, not as a replacement for certified low-level control or safety functions.

## 5. Conclusions

This paper presented a modular active–reactive architecture for mass-customized production with type-3 Asset Administration Shells. Three properties separate it from a conventional split between decision logic and asset data, and Table 11 places them against the related work. The upgrade from type-2 to type-3 is additive: existing submodels keep their structure and their consumers, and the upgrade inserts a pair of contract submodels at the cost of one restart of the reactive part. After that upgrade, the active layer is redeployed, replaced or rolled back with no restart of the reactive layer. And the boundary between the layers is declared in the AAS metamodel itself, as the *ActiveSteps* and *ActiveSkills* submodel templates.

Evaluation used a robotic screwdriving demonstrator built to industrial standards, and not a live production line. It gave a median of 4 s from a call for proposal to the first proposal over 102 answered calls, a median requester activation time of 8 s over 100 activations, and 100 of 100 deployment attempts reaching the negotiation layer. Over a balanced suite of 454 constructed cases, capability checking reached an F1 of 89.6% at USD $7.0\times 10^{-4}$ per evaluation, with a false-positive rate of 10.8% concentrated in the cases whose verdict turns on evidence the provider declaration does not carry.

The practical implication is a migration path. An asset that already exposes a reactive AAS becomes decision-capable by addition, so the modeling investment already made is carried forward instead of rebuilt. Future work should standardize the two submodel templates, without which the contract stays portable within an implementation and not across vendors; extend validation to multi-cell lines with explicit scalability experiments; run the comparison against a monolithic implementation under the design stated in Section 4; harden cybersecurity and governance; and combine semantic decision services with deterministic verification for safety-critical tasks.

## Figures and Tables

**Table 1:** Structure of the *ActiveSteps* and *ActiveSkills* submodel templates. Multiplicity is declared with template qualifiers; nesting is shown by indentation.

Element	Model Type	Mult.	Role
*ActiveSteps* submodel, requester side			
Step	SubmodelElementCollection	1..*n*	One ordered process step of the variant
ReferenceToSubmodel	ReferenceElement	1	Capability submodel the step requires
AllowBidding	Property (boolean)	1	Whether the step is put out to bid
StepStatus	Property (string)	1	Execution state, written back by the active layer
ProductCost	SubmodelElementCollection	1	Cost attributes accumulated for the step
*ActiveSkills* submodel, provider side			
Skills	SubmodelElementCollection	1	Container of the skills offered
Skill	SubmodelElementCollection	1..*n*	One offered skill
ReferenceToSubmodel	ReferenceElement	1	Capability submodel that implements it

**Table 2:** Configurations evaluated during selection of the capability-checking decision core. CE is structural conformity; F1 is computed over the capable/not-capable decision.

Configuration	Model	Cases × Rep.	Accuracy (%)	*CE* (%)	*F*1 (%)
Direct pattern matching	GPT-4.1 Nano	132 × 10	54.40	99.54	65.22
Structured prompt	GPT-4.1 Nano	132 × 10	65.40	99.70	45.87
Structured prompt with deterministic tools	GPT-4.1 Nano	132 × 10	46.50	92.50	58.00
Structured prompt, functional suite	GPT-4.1 Nano	74 × 10	66.40	96.22	74.30
Embedded reasoning	GPT-5	60 × 2	81.70	100.00	81.97
Embedded reasoning, revised submodel	GPT-5	134 × 5	94.60	100.00	88.68
Open-weight reasoning (selected)	GPT-OSS-20B	145 × 5	96.70	100.00	92.00

**Table 3:** Distribution of the 454 decision-quality cases by failure-mode group. Each group is balanced between positive (capable) and negative (not capable) cases. With five repetitions per case the suite yields 2270 evaluations.

Failure-Mode Group	Positive	Negative	Total
G1 — Semantic incompatibility between capabilities	51	51	102
G2 — Insufficient inferential evidence	55	55	110
G3 — Limiting properties and parameterization	53	53	106
G4 — Invalid or inconsistent data	68	68	136
Total	227	227	454

**Table 4:** Comparative performance of the evaluated infrastructures for the capability-checking service. Client-observed times include network transit; the rented GPUs were located in a different continent from the client, so their client-observed times are not comparable with the serverless figure. Server-side times exclude network transit. Generation throughput counts output tokens over generation time and excludes the prefill phase, which neither provider reports.

Metric	Groq (LPU)	RTX 5090	RTX PRO 6000 WS
Unique test cases	145	145	145
Repetitions per case	5	1	1
Test executions	725	145	145
Client-observed time, mean (s)	1.73	5.54	4.83
Client-observed time, s.d. (s)	not recorded	1.50	1.57
Server-side time, mean (s)	not recorded	2.30	not captured
Generation throughput (tokens/s)	893	640	not captured
Infrastructure type	Serverless	Dedicated VPS	Dedicated VPS

**Table 5:** Decision quality of five open-weight reasoning models over the 454 balanced cases, five repetitions each. Group accuracies follow the failure modes of Table 3. Token counts are means per evaluation. Structural conformity was 100% for every model, so it is not repeated per row.

Model	Acc. (%)	*F*1 (%)	G1 (%)	G2 (%)	G3 (%)	G4 (%)	Reas. Tok.
GPT-OSS-20B (deployed)	89.56	89.60	99.02	74.00	97.74	88.68	544
GPT-OSS-120B	89.07	88.99	99.41	74.73	98.68	85.44	567
Qwen3.5 379B A17	88.85	89.18	98.82	73.64	95.47	88.53	1621
Qwen3.5 9B	84.63	85.35	93.33	70.36	90.19	85.29	1804
Qwen3.5 35B A3	70.66	72.34	74.31	58.00	83.02	68.53	3599

**Table 6:** Average token distribution and cost share per capability-checking execution, over the 725 executions of the timing campaign; the token means of the balanced campaign in Table 5 differ slightly because the suite differs. Cost shares are computed at the published rates for the model on the serverless service, USD 0.075 per million input tokens and USD 0.30 per million output tokens, so one output token weighs four input tokens. Shares are given over the price-weighted total of 10,004 input-token equivalents.

Category	Approximate Tokens	Share of Tokens	Share of Cost
Provider submodel	4187	54.42%	41.85%
System prompt	841	10.93%	8.41%
Requester submodel	1896	24.64%	18.95%
Generated answer	138	1.79%	5.52%
Reasoning output	632	8.21%	25.27%
Total input	6924	89.99%	69.21%
Total output	770	10.01%	30.79%
Total	7694	100.00%	100.00%

**Table 7:** Measured operational and deployment metrics from the demonstrator dashboard.

Metric	Symbol	Value	Sample
CFP to first proposal latency (p50/p95)	$t_{cfp}$	4.0/5.0 s	102 CFPs with proposal
Active-layer deploy time (p50/p95)	$t_{deploy}$	8.0/9.0 s	100 successful deploys
CFP throughput during automatic simulation	$\lambda_{cfp}$	1.5718 CFP/min	104 CFPs over 66.1667 min
Deploy success rate	$S_{deploy}$	100.0%	100/100 deploy attempts
Proposal to acceptance latency (p50/p95)	$t_{accept}$	5.0/6.0 s	100 acceptances
CFP to completed service cycle (p50/p95)	$t_{cycle}$	44.0/82.0 s	100 completed services

**Table 8:** Measured resource and decision-quality metrics from the demonstrator and capability-checking campaign. Resource figures are point samples of the active-layer containers attributed to CFP windows; windows shorter than the sampling period contain no sample.

Metric	Symbol	Value	Sample or Note
CPU per CFP (mean/max)	$CPU_{cfp}$	6.37/99.70%	83 of 104 CFP windows contained a sample
Memory per CFP (mean/max)	$MEM_{cfp}$	321.41/587.83 MiB	87 of 104 CFP windows contained a sample
Valid provider–response rate	$Q_{valid}$	91.4%	160 proposals over 175 provider responses
Capability false-positive rate	$FP_{cap}$	10.8%	123 false positives over 1135 negative cases of the balanced suite
Configured product variants	$N_{sku}$	100	product AASs available in BaSyx
Observed product/requester assets	$N_{obs}$	103/100	unique assets/unique requesters in the window

**Table 9:** Measured active-service timing metrics. The provider core orchestrates the capability, feasibility and working services and emits the timing events, so each provider-core figure is the round trip measured from the call to the service that executes the step until its result returns, and not the execution time of the core itself.

Service Timing	Component	p50/p95	Executions
Capability check	provider-core	2.1405/3.1028 s	175
Feasibility check	provider-core	1.4309/1.7677 s	160
Evaluation total	requester-core	0.2965/0.4139 s	100
Evaluation scoring	requester-core	0.0524/0.0924 s	100
Evaluation optimization	requester-core	0.0058/0.0079 s	100
Working service execution	provider-core	34.8794/72.7674 s	100

**Table 10:** Offline equal-share baseline compared with bidding-based provider allocation. The makespan, the throughput and the 38% relative gain are offline estimates of the allocation policy, computed from the measured working-service times of the 100 completed executions. They are not a measured production improvement: the baseline does not re-simulate transport contention, queueing or cell-level disturbances.

Metric	Equal-Share Baseline	Bidding-Based Allocation
Robot assignment counts (R1/R2/R3)	34/33/33	55/26/19
Working-service time, mean	57.48 s	49.18 s
Working-service time, p50/p95	67.13/73.94 s	34.88/72.77 s
Estimated parallel makespan	2440.13 s	1768.22 s
Estimated throughput	147.53 units/h	203.59 units/h
Relative throughput gain	–	38.00%

**Table 11:** Positioning of the proposed architecture against related families of work. *Yes* indicates the property is a reported focus of the family; *Part.* indicates partial or implicit support; *–* indicates it is not addressed. Entries reflect the primary focus reported in the cited works and are not an exhaustive capability audit. The last column records validation on physical hardware, which for the present work is a demonstrator built to industrial standards and not a live production line.

Approach Family	Proactive Type-3	Additive Reactive Preservation	Independent Active Redeploy	Submodel-Level Contract	Physical Validation
In-server/order-driven proactive AAS [7,8]	Yes	–	–	–	Part.
Multi-agent type-3 AAS [9,10,14]	Yes	Part.	Part.	Part.	–
Capability–skill–service composition [11,12,13]	Part.	Part.	–	Yes	Part.
Container-based proactive AAS [19]	Yes	Part.	Yes	–	–
LLM-assisted AAS modeling/inference [22,23,24]	–	–	–	–	–
**This work**	Yes	Yes	Yes	Yes	Yes

## Data Availability

The data presented in this study are available from the corresponding author upon reasonable request. Some data are not publicly available due to industrial confidentiality agreements.

## Abbreviations

AAS	Asset Administration Shell
AI	Artificial Intelligence
CFP	Call for Proposal
HTTP	Hypertext Transfer Protocol
IoT	Internet of Things
LLM	Large Language Model
MQTT	Message Queuing Telemetry Transport
OPC UA	Open Platform Communications Unified Architecture
PPR	Product–Process–Resource

## References

[1] Plattform Industrie 4.0 (2020). Asset Administration Shell Reading Guide. https://www.plattformi40.de/PI40/Redaktion/EN/Downloads/Publikation/Asset_Administration_Shell_Reading_Guide.pdf.

[2] (2023). Asset Administration Shell Specification—Part 1: Metamodel.

[3] Jacoby M., Baumann M., Bischoff T., Mees H., Müller J., Stojanovic L., Volz F. (2023). Open-Source Implementations of the Reactive Asset Administration Shell: A Survey. Sensors.

[4] Alexopoulos A., Kalogeras G., Koutras K., Kalogeras A. (2025). Why Asset Administration Shells: A Survey on Uses and Challenges. IEEE Access.

[5] Abdel-Aty T., Negri E., Galparoli S. (2022). Asset Administration Shell in Manufacturing: Applications and Relationship with Digital Twin. IFAC-PapersOnLine.

[6] Sakurada L., De la Prieta F., Leitão P. (2025). Ten Years of Asset Administration Shell: Developments, Research Opportunities, and Adoption Challenges. IEEE Access.

[7] Grunau S., Redeker M., Göllner D., Wisniewski L., Jasperneite J., Lohweg V. (2022). The Implementation of Proactive Asset Administration Shells: Evaluation of Possibilities and Realization in an Order Driven Production. Kommunikation und Bildverarbeitung in der Automation.

[8] Stolze M., Belyaev A., Kosel C., Diedrich C., Barnard A. Realizing Automated Production Planning via Proactive AAS and Business Process Models. Proceedings of the IEEE International Conference on Emerging Technologies and Factory Automation.

[9] Sakurada L., De la Prieta F., Leitão P. (2025). The Role of Multi-Agent Systems in Realizing Asset Administration Shell Type 3. Future Internet.

[10] Sakurada L., De la Prieta F., Leitão P. (2025). An Agent-Based Approach for Implementing Asset Administration Shell Type 3. IEEE Access.

[11] Simon M., Urban C., Winter M., Köcher A. Realization of a Shared Manufacturing Network Using Capabilities, Skills and Services. Proceedings of the IEEE International Conference on Emerging Technologies and Factory Automation.

[12] Wagner A., Lamoth S., Volkmann M., Hermann J., Ruskowski M. Interaction between FeasibilityCheck, PreconditionCheck and SkillExecution in Skill-Based Machining. In Proceedings of IEEE International Conference on Emerging Technologies and Factory Automation.

[13] Louge T., Namaki Araghi S., Karray M.H., Sarkar A. (2025). Events-Based Semantic Services Composition in Industry 4.0 Using Asset Administration Shell Meta-Model for Digital Twins. Expert Syst. Appl..

[14] Hurtado E., Burgos A., Armentia A., Casquero O. (2025). Self-Configurable Manufacturing Industrial Agents (SMIA): A Standardized Approach for Digitizing Manufacturing Assets. J. Ind. Inf. Integr..

[15] Braunisch N., Heppner S., Garmaev I., Ristin M., Miny T., Kleinert T. Software Evolution of I4.0 Digital Twins with Semantic Patching. Proceedings of the IEEE International Symposium on Industrial Electronics, Ulsan, Republic of Korea.

[16] Nguyen Q.D., Huang Y., Keith F., Leroy C., Thi M.T., Dhouib S. (2024). Manufacturing 4.0: Checking the Feasibility of a Work Cell Using Asset Administration Shell and Physics-Based Three-Dimensional Digital Twins. Machines.

[17] Barnard A. Digital Twin Frameworks: A Comparative Study of AAS, OPC UA, and AML. Proceedings of the International Conference on Electrical, Computer and Energy Technologies.

[18] Neubauer M., Steinle L., Reiff C., Ajdinović S., Klingel L., Lechler A., Verl A. (2023). Architecture for Manufacturing-X: Bringing Asset Administration Shell, Eclipse Dataspace Connector and OPC UA Together. Manuf. Lett..

[19] Ellwein C., Zhang J., Wortmann A., Meckhael A.A.A. A Container-Based Approach for Proactive Asset Administration Shell Digital Twins. Proceedings of the ACM/IEEE International Conference on Model Driven Engineering Languages and Systems.

[20] BMBF—Federal Ministry of Education and Research, Germany BaSyx: The Eclipse Platform for the Digital Twin. https://www.eclipse.org/basyx/.

[21] Sauer C., Eichelberger H. Performance Evaluation of BaSyx Based Asset Administration Shells for Industry 4.0 Applications. Proceedings of the 13th Symposium on Software Performance, Stuttgart, Germany.

[22] Chavez V., Wollert J. (2025). An Industry 4.0 Semantic Framework for the Inference of Generic Field Device Capabilities. IEEE Open J. Ind. Electron. Soc..

[23] Xia Y., Xiao Z., Jazdi N., Weyrich M. (2024). Generation of Asset Administration Shell with Large Language Model Agents: Toward Semantic Interoperability in Digital Twins in the Context of Industry 4.0. IEEE Access.

[24] Shi D., Liedl P., Bauernhansl T. (2024). Interoperable Information Modelling Leveraging Asset Administration Shell and Large Language Model for Quality Control Toward Zero Defect Manufacturing. J. Manuf. Syst..

[25] Jungbluth S., Witton A., Hermann J., Ruskowski M. Architecture for Shared Production Leveraging Asset Administration Shell and Gaia-X. Proceedings of the IEEE International Conference on Industrial Informatics.

